# The benefit of minocycline on negative symptoms in early-phase psychosis in addition to standard care - extent and mechanism (BeneMin): study protocol for a randomised controlled trial

**DOI:** 10.1186/s13063-015-0580-x

**Published:** 2015-03-02

**Authors:** Danuta M Lisiecka, John Suckling, Thomas RE Barnes, Imran B Chaudhry, Paola Dazzan, Nusrat Husain, Peter B Jones, Eileen M Joyce, Stephen M Lawrie, Rachel Upthegrove, Bill Deakin

**Affiliations:** 1 grid.5335.00000000121885934Department of Psychiatry, Behavioural and Clinical Neuroscience Institute, University of Cambridge, Cambridge, UK; 2 grid.5335.00000000121885934Department of Psychiatry, Brain Mapping Unit, University of Cambridge, Herchel Smith Building for Brain and Mind Sciences, Robinson Way, Cambridge, CB2 0SZ UK; 3 grid.450563.10000000404129303Cambridge and Peterborough NHS Foundation Trust, Cambridge, UK; 4 grid.7445.20000000121138111Department of Medicine, Centre for Mental Health, Faculty of Medicine, Imperial College, London, UK; 5 grid.439700.9West London Mental Health NHS Trust, London, UK; 6 grid.5379.80000000121662407Institute of Brain, Behaviour and Mental Health, Clinical and Cognitive Neurosciences, University of Manchester, Manchester, UK; 7 Lancashire Care Early Intervention Service, Accrington, UK; 8 grid.13097.3c0000000123226764Department of Psychosis Studies, Institute of Psychiatry, King’s College, London, UK; 9 grid.5335.00000000121885934Department of Psychiatry, University of Cambridge, Cambridge, UK; 10 grid.83440.3b0000000121901201Institute of Neurology, University College London, London, UK; 11 grid.416119.a0000000098459303Division of Psychiatry, University of Edinburgh, Royal Edinburgh Hospital, Edinburgh, UK; 12 grid.6572.60000000419367486School of Clinical and Experimental Medicine, University of Birmingham, Birmingham, UK; 13 grid.450453.3Early Intervention Service, Birmingham and Solihull Mental Health NHS Foundation Trust, Birmingham, UK; 14 grid.451035.60000000404898305Manchester Mental Health and Social Care Trust, Manchester, UK

**Keywords:** Minocycline, Psychosis, Negative symptoms, Neuroprotection, Double-blind randomised placebo-controlled clinical trial, Multi-centre study, Medication efficacy, Mechanism of action, Blood cytokines screening, Magnetic resonance imaging

## Abstract

**Background:**

Negative symptoms of psychosis do not respond to the traditional therapy with first- or second-generation antipsychotics and are among main causes of a decrease in quality of life observed in individuals suffering from the disorder. Minocycline, a broad-spectrum tetracyclic antibiotic displaying neuroprotective properties has been suggested as a new potential therapy for negative symptoms. In the two previous clinical trials comparing minocycline and placebo, both added to the standard care, patients receiving minocycline showed increased reduction in negative symptoms. Three routes to neuroprotection by minocycline have been identified: neuroprotection against grey matter loss, anti-inflammatory action and stabilisation of glutamate receptors. However, it is not yet certain what the extent of the benefit of minocycline in psychosis is and what its mechanism is. We present a protocol for a multi-centre double-blind randomised placebo-controlled clinical trial entitled The Benefit of Minocycline on Negative Symptoms of Psychosis: Extent and Mechanism (BeneMin).

**Methods:**

After providing informed consent, 226 participants in the early phase of psychosis will be randomised to receive either 100 mg modified-release capsules of minocycline or similar capsules with placebo for 12 months in addition to standard care. The participants will be tested for outcome variables before and after the intervention period. The extent of benefit will be tested via clinical outcome measures, namely the Positive and Negative Syndrome Scale score, social and cognitive functioning scores, antipsychotic medication dose equivalent and level of weight gain. The mechanism of action of minocycline will be tested via blood screening for circulating cytokines and magnetic resonance imaging with three-dimensional T1-weighted rapid gradient-echo, proton density T2-weighted dual echo and T2*-weighted gradient echo planar imaging with N-back task and resting state. Eight research centres in UK and 15 National Health Service Trusts and Health Boards will be involved in recruiting participants, performing the study and analysing the data.

**Discussion:**

The BeneMin trial can inform as to whether in minocycline we have found a new and effective therapy against negative symptoms of psychosis.

The European Union Clinical Trial Register: EudraCT 2010-022463-35 with the registration finalised in July 2011. The recruitment in the trial started in January 2013 with the first patient recruited in March 2013.

## Background

### Negative symptoms in psychosis

Psychosis leads to a significant decrease in a person’s quality of life [1], with negative symptoms contributing the most to impaired functional outcome [2]. Negative symptoms - reduction in emotional and social responsiveness, motivation, speech and movement [3] - respond the least to known antipsychotic treatments among all psychotic symptoms [4]. Their initial severity, together with duration of untreated psychosis (DUP) and cognitive impairments, is the best predictor of an individual’s subsequent impairment in quality of social and occupational functioning [5]. Their positive correlation with DUP links their aetiology to a neuropathic process progressing with continuance of untreated psychosis [6-11]. This hypothesis has led to an interest in neuroprotection during early stages of psychosis as a potential therapeutic target for negative symptoms.

### Efficacy of minocycline for negative symptoms of psychosis

A broad-spectrum tetracyclic antibiotic, minocycline has neuroprotective properties, which could prevent the accumulation of negative symptoms in psychosis [12-15] while improving the prognosis of neurodegenerative diseases and traumatic or ischaemic central nervous system (CNS) insults [16-19]. Due to its lipophilic properties, it has exceptional potential for blood-brain barrier penetration [20]. It is also marked by its relatively low toxicity [21].

Two double-blind randomised placebo-controlled studies of minocycline as a neuroprotective agent for negative symptoms of psychosis have been completed [14,15]. The first was a 2-centre study in Brazil and Pakistan supervised by the University of Manchester with 144 participants [14]. The second, completed in Tel Aviv, Israel, recruited 70 relapsed patients with schizophrenia [15]. Both studies found significant treatment effects on negative symptoms with adjunctive minocycline. In the first trial, significant treatment effects were observed at 6 months, and in the second trial at 3 months. Furthermore, the 2-centre trial demonstrated that therapeutic effects were sustained at 12 months. No other trials investigating the efficacy of minocycline for negative symptoms have been registered in UK or US databases. However, to assess the extent and mechanism of the benefit of minocycline for negative symptoms in psychosis, a trial with a larger cohort is needed.

### Mechanism of action of minocycline on negative symptoms

Although the effectiveness of minocycline in treating negative symptoms has been identified, its mechanism of action remains unresolved [22]. Three possible mechanisms of action have been posited:

1. *Neuroprotection against grey matter loss:* minocycline may directly prevent neurodegeneration of grey matter in individuals with psychosis. Its neuroprotective properties have been shown in stroke [21,23], Parkinson’s disease [24,25], cerebral ischaemia, amyotrophic lateral sclerosis, Huntington’s disease and multiple sclerosis [26]. In psychosis, loss of grey matter occurs early in the course of the disease [27-30], is associated with DUP [31] and predicts diminished functional outcome [32]. By preventing grey matter loss, minocycline may limit negative symptoms.
2. *Anti-inflammatory action:* the anti-inflammatory properties [33,34] of minocycline may prevent neural changes associated with the action of microglia and cytokines in psychosis [35]. The inflammatory response as a mechanism of schizophrenia has been suggested by studies demonstrating microglial activation [35,36]. The gene variants associated with risk of schizophrenia have been linked to increased levels of circulating cytokines [37]. A correlation between negative symptoms and levels of IL-10 cytokine has been observed in untreated psychosis [38]. Increased levels of IL-6 appears in medication-naïve patients with first episode of psychosis [39] and acutely relapsed inpatients [40], whereas elevated levels of IL-6 at the age of 9 doubles the risk of developing psychosis later in life [41]. Minocycline may reduce cytokine levels during psychosis thereby limiting negative symptoms.
3. *Stabilisation of N-methyl-D-aspartate (NMDA) glutamate receptors:* minocycline may improve impaired function of the NMDA glutamate receptor which is thought to display impairment in schizophrenia [42]. Ketamine, which blocks NMDA receptors and causes dysfunctional release of glutamate [43,44], evokes schizophrenia-like negative symptoms in healthy individuals [45]. Minocycline can block ketamine action [46], thus potentially stabilising glutamate release in NMDA receptors [47]. Minocycline may limit negative symptoms either through direct action on glutamate release or through prevention of glutamate neurotoxicity on neuronal branching and glial cells [48]. Although not observable *in vivo*, this can be examined by the association between NMDA receptors and working memory [49,50].

The three mechanisms of action of minocycline may operate separately or jointly. Each represents a potential target for neuroprotectection, effective only when neurodegenerative processes are active and their adverse effects limitable. Thus, minocycline is likely to be observed as being most effective in the early stages of psychosis.

### Benefits and risks of treatment with minocycline

The treatment of psychosis with minocycline may benefit patients by reducing their negative symptoms and, consequently, their difficulties in social and occupational function [5]. This treatment may also reduce the dose of antipsychotic drugs (APDs) necessary to stabilise a patient’s health, thus decreasing potential side-effects of APDs such as weight gain [4]. Finally, new targets for the treatment of psychosis may be discovered [51], which could elucidate the pathophysiology of psychosis and potential biomarkers of increased risk of developing it.

Treatment with minocycline has been associated with vestibular disturbances, gastro-intestinal upset and fatigue [52-54]. Nevertheless, our previous study [14] did not find any such link. Minocycline use has also been associated with the development of a systemic lupus erythematosus - like syndrome [55] with, however, an estimated incidence of 8.8 cases per 100,000 person-years [56]. Patches of discolouration on teeth and skin have also been observed during long-term therapy with minocycline [57,58]. In our previous study [14], three people in each treatment arm reported hyper-pigmentation, but only those in the minocycline arm withdrew from the trial. This was the only noted side-effect withdrawal.

The risks and side-effects observed during minocycline treatment are less severe than those associated with the available antipsychotic drugs, which include extrapyramidal symptoms [59-61], metabolic problems and sedation [62-68]. It is estimated that adding minocycline to standard treatment will have similar effect-size as was observed with a benefit of second-generation APDs over older APDs [62,69].

### Rationale and aims of the BeneMin trial

Finding an effective treatment for negative symptoms in psychosis is of paramount importance due to their role in post-morbid functional outcome [2]. Minocycline has been identified as a potential agent for reducing negative symptoms [14,15]. Therefore, in BeneMin we will investigate the extent and mechanism of action of minocycline on negative symptoms in psychosis. Additionally, we will test whether characteristics such as premorbid and current IQ, DUP and cytokine genotype predict the response to minocycline.

#### Effectiveness hypotheses (EHs)

- EH1) Added to the standard therapy, minocycline administered during the acute phase of psychosis limits development of negative symptoms in comparison to placebo.
- EH2) Minocycline reduces side-effects associated with standard therapy such as weight gain in comparison to placebo.
- EH3) Reduction of negative symptoms leads to improvement in function and life quality in patients treated with minocycline in comparison to placebo.

#### Mechanistic hypotheses (MHs)

- MH1) In comparison to placebo, minocycline reduces grey matter degeneration, which occurs particularly during the early acute phase of the disease. The reduction in grey matter degeneration is observed mainly in the frontal lobes and is associated with reduction of negative symptoms.
- MH2) In comparison to placebo, minocycline diminishes inflammatory action in the CNS thereby reducing negative symptoms. Decreased numbers of circulating cytokines in peripheral blood, and potentially reduced grey and white matter neurodegeneration and micro lesions in the CNS, are observed as the result of minocycline treatment.
- MH3) In comparison to placebo, minocycline improves function of NMDA glutamate receptors and restores optimal levels of glutamate in the CNS, observed through improved cortical function of working memory. The improvement may be a direct result of minocycline action and may cease after the treatment stops or continue through the neuroprotective action of optimal glutamate levels.

## Methods

### Study design

BeneMin is a double-blind, randomised, placebo-controlled clinical trial investigating the extent and mechanism of action of minocycline on negative symptoms of psychosis in addition to standard APD therapy. Participants in an acute psychotic phase will be randomly allocated either to the minocycline or to the placebo arm and continue to receive the assigned treatment for 12 months. Both participants and investigators will be blind to treatment group, which will be guaranteed by a direct data-entry, digital database (OpenCDMS system). Separately, participants will be asked to donate fully-anonymised DNA for genotyping of the interleukin 6 (IL-6) gene [70] and for future investigations. The study will be conducted at eight research centres, and led by the University of Manchester (Table 1).

**Table 1:** **Research centres involved in BeneMin with the corresponding National Health Service (NHS) Trusts, Health Boards and magnetic resonance imaging (MRI) centres**

**Research centres**	**NHS trusts and health boards**	**MRI centres**
University of Manchester	• Manchester Mental Health and Social Care Trust	MRI facility in Salford Royal NHS Foundation Trust
	• Greater Manchester West Mental Health NHS Foundation Trust	
	• Pennine Care NHS Foundation Trust (PIC)	
Lancashire Early Intervention Services	• Lancashire Care NHS Foundation Trust	
Cheshire and Wirral Partnership Early Intervention in Psychosis Service	• Cheshire and Wirral Partnership NHS Foundation Trust	
University of Cambridge	• Cambridge and Peterborough NHS Foundation Trust	Wolfson Brain Imaging Centre Cambridge
	• Norfolk and Suffolk NHS Foundation Trust (PIC)	
University College London	• West London Mental Health NHS Trust	Wellcome Trust Centre for Neuroimaging University College London
	• Central and North West London NHS Foundation Trust	
	• Camden and Islington NHS Foundation Trust	
	• Barnet, Enfield and Haringey Mental Health Trust	
King’s College London	• South London and Maudsley NHS Foundation Trust	Centre for Neuroimaging Sciences King’s College London
University of Edinburgh	• NHS Lothian	Clinical Research Imaging Centre Edinburgh
	• NHS Fife (PIC)	
University of Birmingham	• Birmingham and Solihull Mental Health NHS Foundation Trust	Birmingham University Imaging Centre

PIC, patient identification centre.

The EHs will be tested via clinical outcome variables: levels of negative and positive symptoms of psychosis, observed side-effects and subsequent quality of life. The MHs will be examined via biomarker outcome variables obtained through blood screening for circulating cytokines and magnetic resonance imaging (MRI). In addition to treatment effectiveness, mediators of treatment response such as DUP, cognitive function and IL-6 gene variants will be assessed.

A pre-post treatment design will be used with baseline measurements of the outcome variables taken before commencement of treatment (screening and randomisation visits) and repeated soon after the treatment has finished (month 12 visit). Clinical outcome variables will also be measured between the pre- and post-treatment assessments (month 2, 6 and 9 visits) to capture possible early therapeutic effects of minocycline. The last assessment will be performed 3 months after the participant has finished their treatment (that is 15 months post-randomisation).

### Participants

#### Eligibility criteria

**Figure 1:**
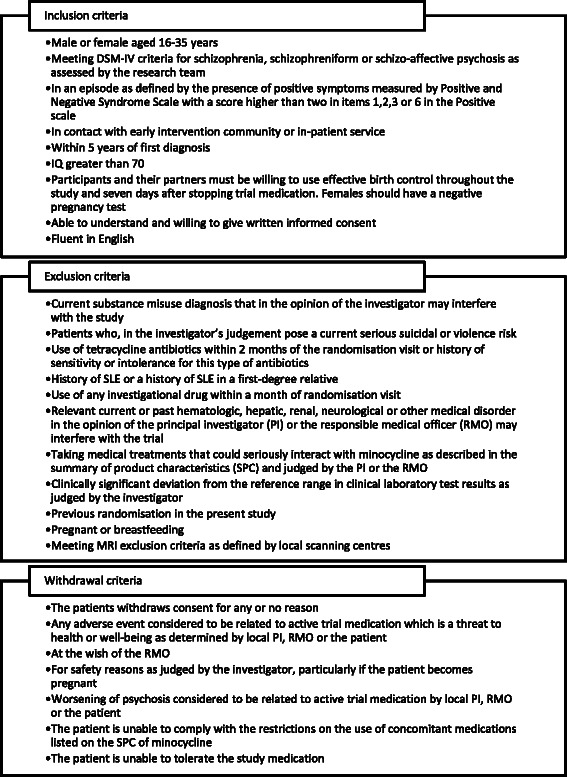
**Eligibility and withdrawal criteria for participants of BeneMin.**

The BeneMin trial will recruit participants in an acute phase of psychosis within 5 years from their first diagnosis. The detailed inclusion and exclusion criteria of the participants are presented in Figure 1.

#### Sample size and recruitment settings

**Figure 2:**
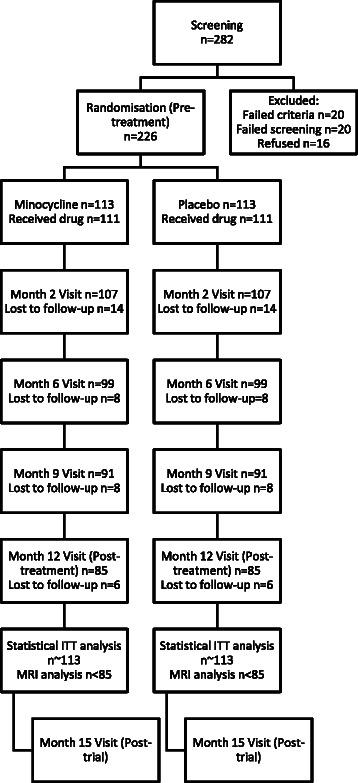
**CONSORT diagram with predicted numbers of participants at each stage of the trial.**

We aim to enrol a total of 226 eligible participants among the 8 research centres. The predicted number of participants remaining in the trial at each stage is presented in the CONSORT diagram in Figure 2. The calculations are based on our previous study of minocycline [14] and a calibration study we performed before this trial [71]. The number of participants was chosen to ensure feasibility of the analysis of post-treatment MRI scans. To meet our recruitment aims we will collaborate with Local Research Networks of the English National Institute for Health Research and Scottish Mental Research Networks. The patients will be recruited via 15 National Health Service (NHS) Trusts and Health Boards (Table 1).

Suitable candidates will first be approached by delegated members of their clinical teams, who will assess each candidate’s eligibility and ask for their permission to be contacted by a project’s research assistant (RA). Eligible candidates will receive the BeneMin patient information leaflet explaining the trial. After expressing interest they will be approached by the RA who will again verify eligibility. Subsequently, the RA will obtain the candidate’s informed consent for participation in the trial, having verified that the candidate fully understands what is involved. Separate consent will be obtained for donating DNA to the bio-bank at the University of Manchester.

#### Withdrawal

At any time a participant can withdraw their consent for participation without giving reasons. The participant will be given an opportunity to discuss the reason for withdrawal and any adverse events (AE). If possible, the participant will be assessed by the investigator. Any potential AE will be followed up by the resident medical officer (RMO). If the participant withdraws their consent at any time beyond 6 months of treatment they will be asked to complete the full post-treatment assessment, followed 3 months later by the post-trial assessment. Their results will be included in the main analysis. The detailed withdrawal criteria are presented in Figure 1.

### Randomisation procedure

After stratification by the research centre, permuted block randomisation integrated with the OpenCDMS system will be used to allocate participants to the treatment arm. After an investigator has requested randomisation for a participant, the OpenCDMS will use the algorithm to allocate the participant to the treatment arm. Subsequently, it will notify a local pharmacy regarding the serial number of a medication kit to use. Participant’s allocation to the study arm will be known only to the OpenCDMS system.

### Planned intervention

Participants will take capsules containing either 100 mg minocycline (modified-release) or matching placebo, 2 per day for the first 2 weeks and then 3 per day for the reminder of the 12-month treatment period in addition to standard therapy. The maximum dose of modified-release minocycline is 3 × 100 mg per 24 hours. Minocycline and placebo will be manufactured, controlled and distributed to the Trusts’ pharmacies by Catalent, Bolton, UK, in accordance with the requirements of the Medicines and Healthcare Products Regulatory Agency (MHRA).

### Clinical outcome measures

We will test hypotheses of minocycline efficacy by assessing changes in primary and secondary clinical outcome measures over treatment.

#### Primary clinical outcome

Severity of negative symptoms of psychosis as measured by the negative symptoms subscale of the Positive and Negative Syndrome Scale (PANSS), an instrument commonly used to assess the efficacy of treatment in schizophrenia [72] consisting of a positive symptoms subscale, a negative symptoms subscale and a general psychopathology subscale [73].

#### Secondary clinical outcomes

1. Body weight and body mass index [74] as a measure of weight gain, a side-effect of the standard APD therapy.
2. Severity of global and positive symptoms of psychosis as measured by the full PANSS and the PANSS positive symptoms subscale.
3. General and social function as measured by:
  Global Assessment of Functioning from the *Diagnostic and Statistical Manual, fourth edition* (DSM-IV) [75,76], a scale assessing psychological, social and occupational function on a hypothetical continuum of health and illness;
  the Social Functioning Scale [77], a self-rating scale assessing social functioning in domains such as social engagement, interpersonal behaviour, pro-social activities, independence or employment.
4. Cognitive function as measured by:
  a short Wechsler Adult Intelligence Scale III for patients with schizophrenia [78], consisting of the information, block design, arithmetic and digit-symbol subtests. This will provide a measure of current IQ and processing speed [79];
  the Wechsler Test of Adult Reading [80], a reading test which estimates premorbid IQ [81];
  Verbal fluency requiring individuals to generate words in response to phonetic or semantic criteria [82];
  the Auditory-Verbal Learning Task [83-85], a test of word list learning and recall.
5. APD dose expressed in chlorpromazine equivalent units [86], assessing the dose of APD required for the patient’s stabilisation.

### Biomarker outcome measures

We will examine the mechanism of action of minocycline by assessing changes in primary and secondary biomarker outcome measures over treatment. The biomarker outcomes will be measured via cytokine blood screening and MRI of the brain.

#### Cytokine blood screening

A blood sample from the participant will be collected into an EDTA tube during a screening visit. Portions of plasma will be separated from the sample for measurement of the cytokine markers associated with inflammatory response in previous studies, namely, IL-6 [87], interleukin-1 receptor antagonist (IL-1RA) [88], monocyte chemotactic protein 1 (MCP-1/CCL2) [89] and C-reactive protein (CRP) [90]. Measurements of IL-6, IL-1RA and MCP-1 will follow a method applied in previous studies of stroke [91,92] and will use multiplex (Luminex®, Manchester, UK) assays. For assessment of CRP level, a sensitive immunoassay will be used [93]. The lowest value among the markers will be classified as the baseline measure of cytokine concentration. Successive blood samples will be collected, and the cytokine tests repeated at month 6, post-treatment (month 12) and post-trial (month 15) visits.

#### Multi-centre magnetic resonance imaging

Three MRI sequences will be performed for each participant pre-treatment (randomisation visit) and post-treatment (12 month visit): three-dimensional T1-weighted rapid gradient-echo (MPRAGE/SPGR); proton density T2-weighted dual echo (PD/T2); and T2*-weighted gradient echo planar imaging (EPI) with N-back task and resting state. MPRAGE/SPGR, a high-resolution structural scan, will be used to assess volume and, thus, neurodegeneration of grey matter. PD/T2 will measure the volume of grey and white matter and of neuro-inflammatory lesions, and will be applied to perform multi-channel texture analysis [94], thus supporting the measurements of neuro-inflammation. EPI N-back via blood-oxygenation-level-dependent (BOLD) contrast will be used to test the neural function associated with the working memory network (dorsolateral-prefrontal, anterior cingulate and parietal cortices) [95] and, thus, NMDA receptors [49,50], whereas EPI resting state will be applied to observe the default network (medial temporal, medial prefrontal and posterior cingulate cortices) [96,97]. Both sequences will be used to examine functional connectivity, and be merged at acquisition into one sequence to test endogenous neural dynamics recovery from cognitive effort [98].

##### N-back task

**Figure 3:**
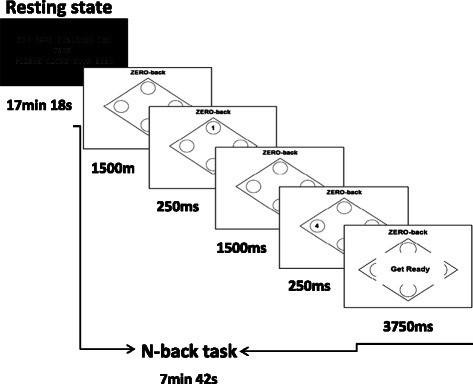
**N-back task and resting state diagram.**

In the N-back task, a well-established method for observing the working memory network [99], the participant presses a button corresponding to the number observed N images previously. The numbers will range from 1 to 4 and the participant will hold a 4-button response box in their right hand. The task will consist of 3 types of blocks, 0-back, 1-back, 2-back, each repeated 5 times in quasi-randomised fashion (the same randomised pattern for each participant). Each block will contain an instruction image (3,750 ms) followed by 15 quasi-randomised trials comprising an image presenting the number (250 ms) and a blank image (1,500 ms) (Figure 3). The response can be given when either the number or the blank image are presented. Reaction times and response accuracy are recorded. After the task has finished an image will appear instructing the participant to close their eyes for the remaining part of the sequence.

##### Multi-centre MRI reproducibility

Multi-centre imaging studies are an effective way to reach a target sample size in a clinical trial within a manageable time-frame. However, due to technical and setting differences between MRI scanners, the question of reproducibility and comparability of data arises. In BeneMin the MRI data will be collected in six MRI centres (Table 1) via six 3T MRI scanners, including two Philips Achieva (Philips Medical Systems, Best, Netherlands), two Siemens Trim Trio, one Siemens Verio (Siemens Medical Systems, Erlangen, Germany) and one GE Signa (General Electric, Milwaukee, WI, USA). Due to technical differences between scanners, a direct replication of scanning parameters would not result in functionally equivalent MRI protocols. Therefore, to develop BeneMin MRI protocols we have applied expertise established by the Alzheimer’s disease neuroimaging initiative (ADNI) [100], a multi-centre imaging project aiming at standardisation of MRI across sites and manufacturers, and conducted a calibration study [71] prior to the trial. The calibration study, in which 12 healthy volunteers were scanned repeatedly in 5 different MRI centres with a protocol similar to ours, showed that between-centre differences accounted for 10% of variation in structural MRI and less than 10% in functional MRI, whereas centre-by-individual interaction was on the level of < 5%. In BeneMin the specifications for each sequence are:

1. *MPRAGE/SPGR:* voxel size of approximately 1 × 1 × 1 mm^3^, phase encode on the anterior-posterior plane, whole brain recorded;
2. *PD/T2:* voxel size of approximately 1 × 1 × 3 mm^3^, phase encode on the right-left plane, whole brain recorded;
3. *EPI:* TR = 2,000 ms, 748 three-dimensional volumes, voxel size of approximately 3 × 3 × 4.5 mm^3^, slices aligned to AC-PC axis, phase encode on the anterior-posterior plane, whole brain recorded.

These parameters have been fixed as their variations cannot easily be compensated during analysis. After these specifications were incorporated, each MRI centre developed the remaining parameters of the local BeneMin MRI sequences based on standardised protocols of ADNI complemented by the centre’s expertise. The protocols thus developed will be used in BeneMin, and are presented in Table 2.

**Table 2:** **Reproducibility and comparability of local BeneMin magnetic resonance imaging (MRI) protocols and N-back presentation**

	**Wolfson Brain Imaging Centre Cambridge**	**MRI facility in Salford Royal NHS Foundation Trust**	**Wellcome Trust Centre for Neuroimaging UCL**	**Centre for Neuroimaging Sciences KCL**	**Clinical Research Imaging Centre Edinburgh**	**Birmingham University Imaging Centre**
MRI scanner	Siemens Trim Trio	Philips Achieva	Siemens Trim Trio	GE	Siemens Verio	Philips Archieva
Field strength (Tesla)	3 T	3 T	3 T	3 T	3 T	3 T
Head Coil	12-channel	8-channel	12-channel	12-channel	12-channel	8-channel
MPRAGE/SPGR time	9:14	10:52	9:14	10:19	9:14	10:52
PD/T2 time	5:08	5:33	5:08	4:49	5:08	5:33
EPI time	25:02	25:04	25:00	25:04	25:02	25:08
MPRAGE/SPGR voxel size FH/IS (mm)	1	1	1	1	1	1
MPRAGE/SPGR voxel size AP (mm)	1	1	1	1	1	1
MPRAGE/SPGR voxel size RL (mm)	1	1.2	1.2	1.2	1.2	1.2
PD/T2 voxel size AP (mm)	0.9	0.9	0.9	0.9	0.9	0.9
PD/T2 voxel size RL (mm)	0.9	0.9	0.9	0.9	0.9	0.9
PD/T2 voxel size FH/IS (mm)	3	3	3	3	3	3
EPI voxel size RL (mm)	3	3	3	3.3	3.4	3
EPI voxel size AP (mm)	3	3	3	3.3	3.4	3
EPI voxel size FH/IS (mm)	4.5	4.5	4.5	4.5	4	4.5
FOV MPRAGE/SPGR FH/IS (mm)	256	256	256	260	256	256
FOV MPRAGE/SPGR AP (mm)	256 (240)	256 (240)	256 (240)	260	256 (240)	240
FOV MPRAGE/SPGR RL (mm)	192	204	192	204	176	204
FOV PD/T2 AP (mm)	240	240	240	240	240	240
FOV PD/T2 RL (mm)	240 (214)	240 (210)	240 (214)	240	240 (214)	210
FOV PD/T2 FH/IS (mm)	144	144	144	144	144	144
FOV EPI RL (mm)	192	192	192	211	220	192
FOV EPI AP (mm)	192	192	192	211	220	192
FOV EPI FH/IS (mm)	126	175.5	126	126	129	175.5
Slice plane MPRAGE/SPGR	Sagittal	Sagittal	Sagittal	Sagittal	Sagittal	Sagittal
Slice plane PD/T2	Transverse	Transverse	Transverse	Transverse/axial	Transverse	Transverse
Slice plane EPI	Transverse	Transverse	Transverse	Transverse/axial	Transverse > C-19.8 > S0.8	Transverse
EPI acquisition	Interleaved	Interleaved	Interleaved	Interleaved	Interleaved	Interleaved
Number of slices for MPRAGE/SPGR	176	170	160	170	160	170
Number of slices for PD/T2	48	48	48	48	48	48
Number of slices for EPI	28	39	28	28	26	39
Number of volumes for EPI	748	748	748	748	748	748
Fold-over/phase encoding direction MPRAGE/SPGR	A > > P	A > > P	A > > P	P > > A	A > > P	A > > P
Fold-over/phase encoding direction PD/T2	R > > L	R > > L	R > > L	L > > R	R > > L	R > > L
Fold-over/phase encoding direction EPI	A > > P	A > > P	A > > P	P > > A	A > > P	A > > P
TE MPRAGE/SPGR (ms)	2.98	Shortest (3.1)	2.91	Min Full	2.98	3.1
TR PD/T2 (ms)	3,000	3,000	3,000	3,000	3,000	3,000
TE 1 PD/T2 (ms)	11	Shortest (10)	11	Min Full	11	Shortest (10)
TE 2 PD/T2 (ms)	99	96	99	97.2	101	96
TR EPI (ms)	2,000	2,000	2,000	2,000	2,000	2,000
TE EPI (ms)	30	30	30	30	26	30
Flip angle MPRAGE/SPGR (deg)	9	8	9	8	9	8
Flip angle EPI (deg)	90	79	90	90	66	79
Distance factor for EPI (%)	0	0	0	0	25 (1 mm)	0
MRI data format	DICOM	DICOM	DICOM	DICOM	DICOM	DICOM
N-back task software	Visual studio	E-Prime 2.0.10	E-Prime 2.0.10	E-Prime 2.0.10 Professional	E-Prime 2.0.10	E-Prime 2.0.10 Professional
Response pad	4-button right handed	4-button right handed	4-button right handed	4-button right handed	4-button right handed	5-button right handed
Response box connection	Parallel port	USB	USB	USB	USB	USB
TTL pulses box connection	Parallel port	USB	USB	USB	USB	Parallel port
N-back data format	TXT	TXT	TXT	TXT	TXT	TXT

NHS, National Health Service; UCL, University College London; KCL, King’s College London; GE, General Electric; FH/IS, foot-to-head/inferior-to-superior; AP, anterior-to-posterior; RL, right-to-left; FOV, field of view; A > > P, from anterior to posterior; P > > A, from posterior to anterior; R > > L, from right to left; L > > R, – from left to right; TE, echo time; TR, repetition time; TTL, transistor-transistor logic.

#### Primary biomarker outcomes

1. Medical prefrontal grey matter volume (MH1).
2. Circulating cytokine IL-6 concentration (MH2).
3. Dorsolateral-prefrontal cortex BOLD response, accuracy and functional connectivity in N-back task (MH3).

#### Secondary biomarker outcomes

1. Total and other regional grey matter volumes (MH1).
2. Volume and multi-channel features of grey and white matter as measured by PD/T2 and of neuro-inflammatory lesions, and concentration of the remaining cytokine markers (MH2).
3. Functional connectivity and the distribution of Hurst exponent of fMRI noise in resting state (MH3).

### Side-effects and adverse events

Possible side-effects and potential co-morbidities will be recorded. Depression will be assessed with self-rating Calgary Depression Scale for Schizophrenia [101], extrapyramidal symptoms with the Simpson-Angus Scale [102], the Barnes Akathisia Rating Scale [103], the Abnormal Involuntary Movements Scale [104] and APD subjective side-effects with the Antipsychotic Non-Neurological Side-Effects Rating Scale [105]. The seven-point adherence to treatment scale will be also used [106]. Additionally, any AE such as occurrence of an undesirable medical condition or deterioration of an existing medical concern will be recorded throughout the treatment duration and the post-trial period.

### Assessment schedule

Each participant will undergo seven assessment sessions, which will include the screening, the pre- and post-treatment assessments, and the sessions between onset and completion of treatment. The detailed measurements taken during each session are presented in Table 3.

**Table 3:** **Purpose and schedule of assessments in BeneMin**

	**Screening**	**Randomisation (Pre-treatment)**	**Month 2**	**Month 6**	**Month 9**	**Month 12 (Post-treatment)**	**Month 15 (Post-trial)**
Inclusion and exclusion criteria checklist	EM						
Case-note diagnostic checklist	EM						
Diagnostic and eligibility checklist	EM and CO					CO	
Mini-International Neuropsychiatric Interview	EM and CO					CO	
Positive and Negative Syndrome Scale	EM and CO	CO	CO	CO	CO	CO	CO
Wechsler Adult Intelligence Scale III; 4 subtest short form	EM and CO					CO	CO
Wechsler Test of Adult Reading	EM and CO						
Verbal fluency	EM and CO					CO	CO
Auditory-Verbal Learning Task	EM and CO					CO	CO
Medication treatment history	EM and CO	CO	CO	CO	CO	CO	CO
Pregnancy urine dipstick test	EM	WCM	WCM	WCM	WCM	WCM	
Drug urine screening test	EM			WCM			
Drug use questionnaire	EM	WCM	WCM	WCM	WCM	WCM	SCo
Blood laboratory screening for kidney and liver function	EM					WCM	
Blood pressure and heart rate	EM					WCM	SCo
MRI screening questionnaire	EM						
Duration of untreated psychosis	RP						
Body weight and body mass index		CO				CO	CO
Global Assessment of Functioning		CO	CO	CO	CO	CO	CO
Social Functioning Scale		CO		CO		CO	CO
MRI scanning		BO				BO	
Blood cytokine screen test		BO		BO		BO	BO
Saliva Oragene kit for DNA donation		RP					
Withdrawal criteria checklist		WCM	WCM	WCM	WCM	WCM	
Calgary Depression Scale for Schizophrenia		SCo	SCo	SCo	SCo	SCo	SCo
Simpson and Angus Scale		SCo		SCo		SCo	SCo
Barnes Akathisia Scale		SCo		SCo		SCo	SCo
Abnormal Involuntary Movements Scale		SCo		SCo		SCo	SCo
Antipsychotic Non-Neurological Side-Effects Rating Scale		SCo	SCo	SCo	SCo	SCo	SCo
7-point compliance scale			AT	AT	AT	AT	

EM, eligibility measure; CO, clinical outcome; RP, response predictor; BO, biomarker outcome; WCM, withdrawal criteria measure; SCo, side-effects and co-morbidity; AT, adherence to treatment.

Intersections between lines and columns show if the test took place during the visit (cells containing an abbreviation). Abbreviations explicate the reason why the test was performed at a particular stage of the trial and how the particular measure fits into statistical design of the study.

### Analysis

#### Statistical analysis

A single round of analysis will be undertaken after all outcome results have been collected. Treatment effects will be assessed using the intention-to-treat method unless a significant amount of non-adherence to treatment is observed, in which case the complier-average causal effect method will be applied. In both cases non-adherence will be accounted for, since both methods allow for missing data via either inverse probability weights [107,108] or maximum likelihood estimation [109,110], and non-adherence is likely to correlate with withdrawals. Group differences in outcomes and mediators of treatment response will be assessed via a random effects model for longitudinal data, whereas MHs will be tested with instrumental variable methods [111].

#### MRI analysis

All MRI scans will be visually inspected for quality and possible acquisition artefacts. Subsequently, each scan will undergo standardised modality-specific temporal and spatial pre-processing:

1. *MPRAGE/SPGR and PD/T2:* brain tissue extraction, segmentation via scan-specific tissue priors, non-linear registration to the Montreal Neurological Institute 152 (MNI) standard space [112], smoothing with an isotropic Gaussian kernel;
2. *EPI:* motion and slice-timing correction, brain tissue extraction, non-linear co-registration with MNI-registered MPRAGE/SPGR, smoothing with an isotropic Gaussian kernel, intensity normalisation, high pass temporal filter.

For both MPRAGE/SPGR and PD/T2, voxel-based morphometry analysis will be performed [113], with additional lesion count in PD/T2 [114]. Texture features identified as neuro-inflammation markers in multiple sclerosis [94] will be measured in PD/T2. In EPI a general linear model will be used to model the BOLD signal [115]. Additionally, for the EPI resting state, the Hurst exponent will be extracted [116]. Group comparisons for all measures will be performed for the whole brain and for the regions of interest defined as belonging to positive and negative task networks [95-97] and located via MNI Atlases [117]. A mixed effects model for repeated measures will be used to evaluate treatment effectiveness defined as interaction between treatment arm and time. Additionally, a partial least squares model will be applied to identify brain structures and activation patterns associated with the extent of symptomatic change. Furthermore, seed-based correlations of voxel time-series will be used in EPI to establish treatment effects on functional connectivity of the positive and negative task networks [118]. All MRI analyses will be performed via non-parametric testing, with appropriate corrections for multiple comparisons [119].

### Ethics approval and research sponsor

The study design and the described procedures have been approved by the National Research Ethics Service Committee North West - Greater Manchester Central (REC reference number: 11/NW/0218). The following local Research and Development departments of NHS Trusts and Health Boards after being presented with the aforementioned approval agreed to host the study: the Manchester Mental Health and Social Care Trust Research and Innovation Office, the Greater Manchester West Mental Health NHS Foundation Trust Research and Development Office, the Pennine Care NHS Foundation Trust Quality Assurance, Research and Innovation Unit, the Lancashire Care NHS Foundation Trust Research Department, the Cheshire and Wirral Partnership NHS Foundation Trust Academic Unit, the Cambridgeshire and Peterborough NHS Foundation Trust Research and Development Department, the Norfolk and Suffolk NHS Foundation Trust Research and Development Office, the UCL/UCLH Joint Research Office, the North Central London Research Consortium, the South London and Maudsley/IoPPN Research and Development Office, the NHS Lothian Academic and Clinical Central Office for Research and Development, the NHS Fife Research and Development Resource Centre and the Birmingham and Solihull Mental Health Foundation NHS Trust Research and Development Department. The Manchester Mental Health and Social Care Trust is the research sponsor and will be responsible for monitoring, audit and pharmacovigilance of the trial. The trial has the approval of MHRA.

## Discussion

Minocycline has the potential to improve the treatment of negative symptoms in psychosis and thereby protect premorbid levels of functioning and consequently the quality of life for many patients. The medication has low toxicity and is not expensive to manufacture, hence it could be a straightforward addition to standard care. Furthermore, minocycline may reduce the dose of antipsychotic drugs necessary to stabilise a patient’s condition and thus limit side-effects associated with standard antipsychotic therapy.

Therapy with minocycline can potentially prevent irreparable neurodegenerative changes observed in the early stages of psychosis, which are proposed as a cause of negative symptoms. If the mechanism of action of minocycline is revealed, new targets for therapy of psychosis will be established and novel medications with clinical properties associated with the mechanism may be developed. Discovering the mechanism could also elucidate a neural basis of psychosis itself.

BeneMin as a multi-site clinical trial will evaluate the extent and mechanism of efficacy of minocycline for negative symptoms in a manageable time-frame. However, the multi-site character of BeneMin raises an issue of reproducibility and comparability of the data. Therefore, standardisation of procedures such as MRI and compatible training for the data collectors will be of key importance. On the other hand, recruiting participants from many geographical locations increases ecological validity of the sample which in turn helps with generalisation of the findings.

## Trial status

The trial is ongoing. Participants are currently being recruited.

## Abbreviations

ADNI: Alzheimer’s disease neuroimaging initiative
AE: adverse event
APD: antipsychotic drug
BOLD: blood-oxygenation-level-dependent
CNS: central nervous system
CRP: C-reactive protein
DSM-IV: *Diagnostic and Statistical Manual, fourth edition*
DUP: days of untreated psychosis
EHs: effectiveness hypotheses
EPI: echo planar imaging
IL-1RA: interleukin-1 receptor antagonist
IL-6: interleukin 6
MCP-1: monocyte chemotactic protein 1
MHs: mechanisitic hypotheses
MHRA: The Medicine and Healthcare Products Regulatory Agency
MNI: Montreal Neurological Institute
MRI: magnetic resonance imaging
NHS: The National Health Service
NMDA: N-methyl-D-aspartate
PANSS: The Positive and Negative Syndrome Scale
PIC: patient information centre
RA: research assistant
RMO: resident medical officer
